# *De novo* assembly of a potential fish pathogen *Lactococcus lactis* IFLLLBEK1 isolated from *Labeo bata*

**DOI:** 10.1128/mra.00455-25

**Published:** 2025-06-23

**Authors:** Basanta Kumar Das, Vikash Kumar, Suvra Roy, Debasmita Mohanty, Kampan Bisai, Hirak Jyoti Chakraborty

**Affiliations:** 1 ICAR-Central Inland Fisheries Research Institute 235412 , Barrackpore, Kolkata, India; California State University San Marcos, San Marcos, California, USA

**Keywords:** *Lactococcus lactis*, *Labeo bata*, fish pathogen, *de novo* assembly

## Abstract

We report the complete genome sequence of *Lactococcus lactis*, a bacterium (Streptococcaceae, Lactobacillales) isolated from infected *Labeo bata* in 2023. The *L. lactis* IFLLLBEK1 strain (chromosome CP169315 and plasmid CP169316) has a 2.56 Mb genome size, 2,425 coding gene sequences, 7, 6, 6 (5S, 16S, and 23S) rRNA, 65 tRNA, 4 ncRNAs, and 1 strict antimicrobial resistance gene in the glycopeptide antibiotic class.

## ANNOUNCEMENT

*Lactococcus lactis*, a gram-positive bacterium (Family Streptococcaceae) ubiquitously found in aquatic and terrestrial environments, is characterized as a non-motile and non-spore-forming bacterium (1–3). *L. lactis* is known to play a commercially important role in feed fortification, milk fermentation, and vaccine production, but pathogenic *L. lactis* has been isolated from many clinical cases in recent years (2). In this study, the *L. lactis* IFLLLBEK1 strain (chromosome CP169315 and plasmid CP169316) was isolated from Indian minor carp bata (*Labeo bata*) cultured in Chalk Bherry, located in the East Kolkata Wetland (latitude: 22°55′ N and longitude: 88°44′ E), West Bengal, India. The symptomatic fish (lethargy, loss of appetite, deep ulceration, and hemorrhage on the ventral body surface) tissue samples were homogenized aseptically (BeadBlaster, Sigma-Aldrich) and were serially diluted using sterile 1× PBS, and 100 µL of each dilution was spread onto tryptone soya agar (TSA) Petri dishes and incubated at 28°C for 18–20 h (4). Afterward, based on uniqueness in morphology, size, and color, a single colony was picked from TSA plates and cultured overnight in TSB at 28°C. The isolate was characterized based on *in vitro* (biofilm [5] and swimming motility assay [6]) and *in vivo* methods (survival assay [7, 8]). The results showed that *L. lactis* exhibited significantly higher biofilm and swimming motility activity and induced 100% mortality within 24 h post-injection in *L. bata*.

Bacteria were grown in TSA at 28°C under aerobic conditions for 24 hours. The colonies were scraped, suspended in TE buffer, and pelleted by centrifugation. Genomic DNA was extracted from the pellet using a Qiagen DNeasy PowerSoil Pro Kit (Cat. No: 47014). DNA shearing (Megaruptor 3, Belgium), sheared DNA size selection (8–10 kb), SMRTbell Library size distribution (Agilent FEMTO pulse, Agilent Technologies, USA), SMRTbell Library preparation (Template Prepkit 3.0), SMRT Library purification, library size selection to remove <5 kb fragments, and preparation of bound complex (Pacific Biosciences, USA) were performed for sequencing. *De novo* whole-genome sequencing was performed on the PacBio Sequel II (Nucleome Informatics Pvt Ltd, Pacific Biosciences, USA). The generated raw subreads were converted to HiFi reads using CCS (version 6.2.0) (9). The genome assembly and assessment were done using Flyev2.9.3, a *de novo* assembler for single-molecule sequencing reads using repeat graphs (10). Then, genome completeness was done using the tools QUAST (version 5.3) and BUSCO (version 5.8.2) using bacteria_odb10 as lineage (11). Later, annotation was done using the Prokaryotic Genome Annotation Pipeline (PGAP) of NCBI (version 6.8) (12). Average nucleotide identity (ANI) analysis by PGAP with the previously submitted reference genome of *L. lactis* revealed a genomic similarity of 98.07%. The Resistance Gene Identifier tool (version 6.0.3) was used to identify antimicrobial resistance (AMR) genes using reference data from the Comprehensive Antibiotic Resistance Database (version 4.0.0) (13). A whole-genome phylogeny was constructed using CLC Genomics Workbench 12. The k-mer-based tree was built using a neighbor-joining approach, and evolutionary distance was measured using Mahalanobis’ methods.

**Fig 1:**
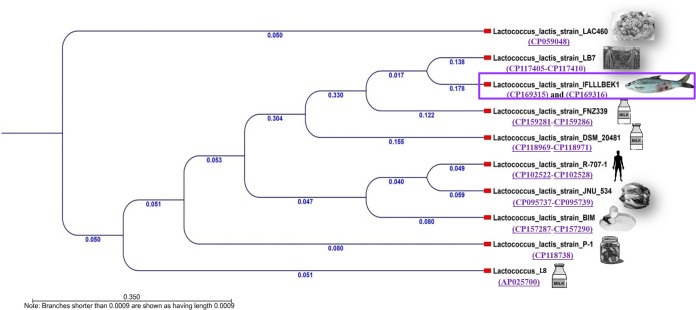
The phylogenetic tree comprises isolated *L. lactis* and strains from different sources and countries. This figure was generated using the CLC Genomics Workbench, and the k-mer-based tree was built using a neighbor-joining approach and Mahalanobis’ methods. The phylogenetic tree demonstrates that the isolated strain formed a clade with fermented rice, faba beans, cheese starter culture, kimchi, rhizosphere, milk, and fermented pepper isolates from Finland, Belarus, South Korea, Japan, Italy, China, and New Zealand.

The genomic assembly statistics of *L. lactis* (chromosome CP169315 and plasmid CP169316) are provided in Table 1. The assembled genome in this study was 99.29% complete and 0.5% contaminated. After assembly, two contigs were identified as complete circular sequences (Bandage, version 0.9.0) of 2,566,861 bp, with a G + C content of 35% and 34%. We identified one AMR gene in the glycopeptide antibiotic class. The *L. lactis* IFLLLBEK1 strain sequence was aligned with the genome sequences of the most commonly identified *L. lactis* strains retrieved from NCBI GenBank, and a phylogenetic tree was constructed (Fig. 1).

**TABLE 1:** Complete genome sequence information of *Lactococcus lactis* isolated from fish species

Bacterial isolate	*Lactococcus lactis* IFLLLBEK1
Sequencing technology and assembly method	PacBio Sequel II version v11.0
Raw reads number	146.7 M
Average length	1,283,430.5
No. of contigs	2
Total length of contigs (bp)	2,566,861
Chromosome length (bp) (contig 1)	2,498,656 (GC% 35)
Plasmid length (bp) (contig 2)	68,205 (GC% 34)
*N*_50_	2.5 Mb
Genome coverage	35.04×
Completeness	99.29%
Contamination	0.5%
Genes (total)	2,548
Protein-coding genes	2,425
No. of rRNAs	7, 6, 6 (5S, 16S, 23S)
No. of tRNAs	65
No. of ncRNAs	4
Antimicrobial resistance genes	CP169315 (Perfect—0, Strict—1, and Loose—149) and CP169316 (Perfect—0, Strict—0, and Loose—4)
ANI	98.07% (reference genome accession number: CP065736 and CP065737)
BioProject number	PRJNA1152940
BioSample accession no.	SAMN43381642
Accession numbers	CP169315 and CP169316

## Data Availability

The whole-genome sequence of IFLLLBEK1 is available in GenBank under accession numbers CP169315 and CP169316. The BioSample and BioProject accession numbers are SAMN43381642 and PRJNA1152940, respectively. The raw sequence data have been deposited in the Sequence Read Archive under accession numbers SRR30906685. The RGI (CARD) analysis uploaded in Figshare and the data set can be accessed in https://figshare.com/articles/journal_contribution/Resistance_Gene_Identification_RGI_analysis_files_for_i_Lactococcus_lactis_i_IFLLLBEK1/28888790.
